# Multiple Neonatal Teeth in a Preterm Neonate: A Case Report

**DOI:** 10.31729/jnma.5611

**Published:** 2021-12-31

**Authors:** Uttara Gautam, Rajan Phuyal, Abhin Sapkota, Vijaya Kumar Chikanbanjar

**Affiliations:** 1 Department of Pediatrics, Kathmandu Medicai College Teaching Hospital, Sinamangal, Kathmandu, Nepal

**Keywords:** *neonate*, *preterm infants*, *tooth eruption*

## Abstract

Prematurity and low birth weight are significantly associated with delayed dentition. Few cases of the eruption of a tooth immediately at or after birth in preterm neonates have been reported in the literature, although this is a rare presentation. The aetiology of this rare biological aberration is yet to be established but has been associated with hereditary predilection, nutritional factor, endocrine causes, infection, and some syndromes. Here, we present a case of a preterm male neonate at 28 weeks of gestation with very low birth weight and respiratory distress who presented with three neonatal teeth, two maxillary central incisors, and one mandibular central incisor and its subsequent management.

## INTRODUCTION

Neonatal teeth are defined as teeth present below 30 days of birth and natal teeth as teeth present at birth.^1,2^ They usually occur in the incisor and molar regions of the mandibular arch with natal teeth being three times more common than neonatal teeth.^3,4^ In newborns with low birth weight or gestational age below 30 weeks, a significant delay in eruption of the first deciduous tooth has been shown.^5^ Natal or neonatal teeth in preterm neonates are rare with only a few mentions in the literature.^6–9^ Here, we report multiple neonatal teeth in a preterm neonate of 28 weeks of gestation.

## CASE REPORT

A single, live, preterm male was born at 28 weeks of gestation via emergency lower segment cesarean section to a primigravida mother for antepartum hemorrhage and premature prolonged rupture of membrane for 12 days with APGAR of 8/10 and 9/10 at one and five minutes respectively and birth weight of 1.25kg (very low birth weight).

After birth, the baby was admitted to the neonatal intensive care unit, where he developed signs of respiratory distress for which he was managed with Mechanical Continuous Positive Airway Pressure (CPAP) and intravenous (IV) antibiotics. A septic workup was carried out. Dobutamine injection at 10mcg/kg/min was started for cardiovascular instability and injection caffeine citrate 20mg/kg as loading dose and 10mg/kg/day as maintenance dose was provided for apnoea of prematurity. Inotrope support was removed and feeding was started on the 4th day of life. Multivitamin and calcium supplementation was started on the 7th day of life. Dexamethasone therapy as per low dose DART regimen was instituted because of evolving bronchopulmonary dysplasia on the 14th day of life. The baby was weaned from mechanical CPAP to nasal prong on the 16th day and room air on the 19th day of life. Kangaroo mother care was initiated on the 20th postnatal day.

**Figure 1:**
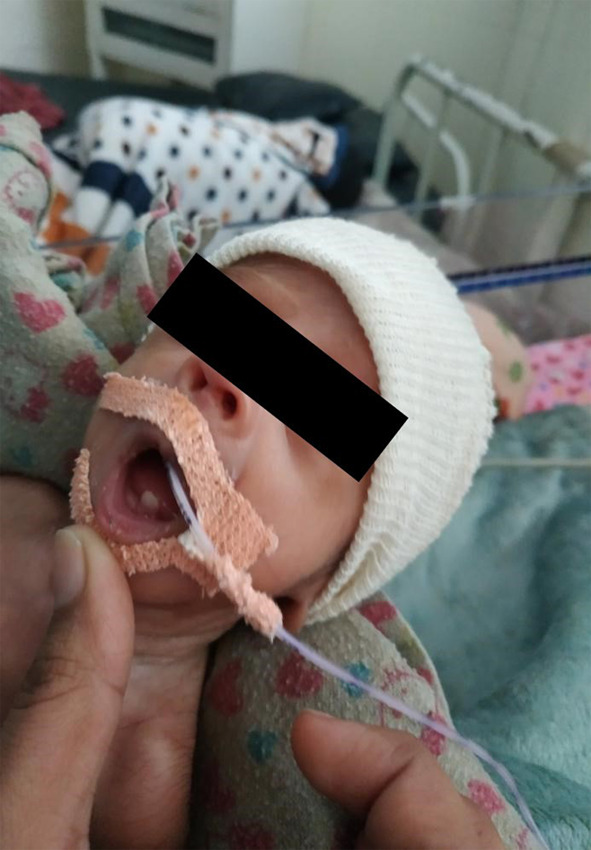
Mandibular central incisors.

**Figure 2:**
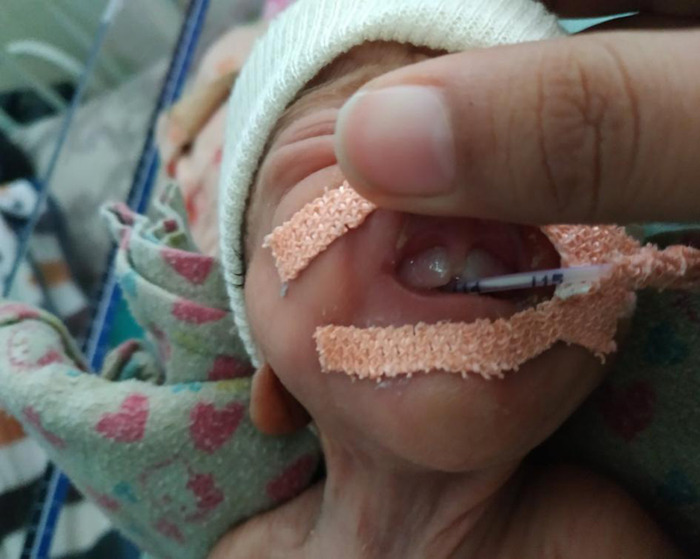
Maxillary central incisors.

On 21st post-natal day, three neonatal teeth, two maxillary central incisors, and one mandibular central incisor were observed. At that time, the corrected gestational age of the baby was 31 weeks and 3 days of gestation (Figure 1 and 2).

The lower left central incisor was small in size, conical, and whitish exhibiting grade three mobility. Lips, gingiva, tongue, palate, buccal mucosa, and floor of the mouth were normal. In contrast, maxillary central incisors were immobile. No lymphadenopathy was exhibited.

A decision to extract a lower incisor for the risk of aspiration was made. It was a shell-shaped crown fixed poorly to the alveolus by gingival tissue. No root was present. Upper central incisors were decided to be left in place. One day after the extraction of the lower incisor a fleshy mass was seen in its place which was soft and mobile. At the time of discharge, the baby was hemodynamically stable. The mandibular soft tissue mass was persistent and the upper central incisors remained immobile.

## DISCUSSION

The occurrence of natal and neonatal teeth in preterm, more so, in very preterm (less than 32 weeks of gestation) and extremely preterm (less than 28 weeks of gestation) is extremely rare. The presence of such teeth is frequently associated with several syndromes and other anomalies. These include Ellis-van Creveld syndrome, Hallerman-Streiff syndrome, Rubinstein-Taybi syndrome, Pierre-Robin syndrome, cyclopia, Pallister-Hall syndrome, short rib-polydactyly type II, cleft lip and palate, Pfeiffer syndrome, Ectodermal dysplasia, craniofacial dysostosis, Sotos syndrome, and epidermolysis bullosa simplex, etc.^10,11^ No such syndromes or anomalies were identified in this case.

While there are no clearly established gender differences in prevalence, some authors have found slight predilection for females while others say that no such difference exists.^12^ Baby was a male gender in our case.

Although the aetiology of this condition remains unclear, several factors like the superficial position of the tooth germ, hormonal stimulation, infection or malnutrition have been found to play an important role.^13^ Hereditary transmission, osteoblastic activity inside the germ area, and hypovitaminosis are also other postulated theories.^6,7^

Classification of natal teeth in the literature is divided into four clinical categories:

1. Shell-shaped crown fixed poorly to the alveolus by gingival tissue and absence of a root.
2. Solid crown fixed poorly to the alveolus by gingival tissue and little or no root.
3. Eruption of crown incisal margin through the gingival tissues.
4. Oedematous gingival tissue with an unerupted but palpable tooth.^14^

This case falls under the first category where only a shell-shaped crown was poorly fixed to underlying alveolar gingiva with absent root.

Natal and neonatal teeth in a neonate is a biological rarity posing a challenge to pediatricians and dentists where the individual case should be carefully evaluated to decide whether to keep or extract the teeth. The decision to extract teeth is based on factors like the grade of mobility, development of teeth, feeding difficulties, the clinical status of the baby, and the risk of aspiration. In this case, the lower left central incisor was exhibiting grade three mobility and was decided to be extracted due to the risk of aspiration whereas the two upper central incisors were decided to be left in place based on their low grade of mobility. Further studies are required to understand the aetiology of premature teeth eruption in a term as well as preterm neonates.
